# 4-Bromo-*N*-(3,4,5-trimethoxy­benzyl­idene)aniline

**DOI:** 10.1107/S1600536809003432

**Published:** 2009-01-31

**Authors:** Aliakbar Dehno Khalaji, Matthias Weil, Kazuma Gotoh, Hiroyuki Ishida

**Affiliations:** a Department of Chemistry, Faculty of Science, Golestan University, Gorgan, Iran; b Institute for Chemical Technology and Analytics, Division of Structural Chemistry, Vienna University of Technology, Getreidemarkt 9/164-SC, A-1060 Vienna, Austria; c Department of Chemistry, Faculty of Science, Okayama University, Okayama 700-8530, Japan

## Abstract

The title compound, C_16_H_16_BrNO_3_, adopts an *E* configuration with respect to the imine C=N bond. The two benzene rings are twisted with respect to each other at an angle of 38.3 (1)°. In the crystal structure, mol­ecules are connected by weak bifurcated C—H⋯(O, O) hydrogen bonds, forming a helical chain along the *b* axis.

## Related literature

The structure of the isotypic 4-chloro compound was reported by Khalaji *et al.* (2009 ▶). For structures containing a 4-bromo­aniline unit, see: Khalaji *et al.* (2007 ▶); Khalaji & Harrison (2008 ▶).
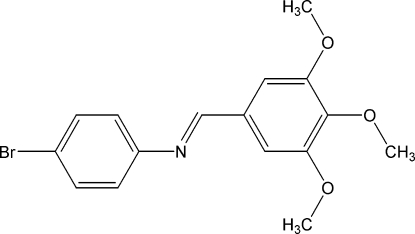


## Experimental

### 

#### Crystal data

C_16_H_16_BrNO_3_
*M* *_r_* = 350.21
Monoclinic, 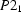

*a* = 7.1951 (4) Å
*b* = 8.3722 (5) Å
*c* = 13.2882 (8) Å
β = 104.413 (3)°
*V* = 775.27 (8) Å^3^
*Z* = 2
Mo *K*α radiation
μ = 2.66 mm^−1^
*T* = 296 (2) K
0.40 × 0.30 × 0.15 mm

#### Data collection

Bruker APEXII CCD diffractometer
Absorption correction: multi-scan (**SADABS**; Bruker, 2006 ▶) *T*_min_ = 0.403, *T*_max_ = 0.671
18229 measured reflections
3497 independent reflections
3064 reflections with *I* > 2σ(*I*)
*R*_int_ = 0.023

#### Refinement

*R*[*F* ^2^ > 2σ(*F* ^2^)] = 0.025
*wR*(*F* ^2^) = 0.065
*S* = 1.09
3497 reflections
193 parameters
1 restraint
H-atom parameters constrained
Δρ_max_ = 0.30 e Å^−3^
Δρ_min_ = −0.53 e Å^−3^
Absolute structure: Flack (1983 ▶), 1511 Friedel pairs
Flack parameter: 0.012 (6)

### 

Data collection: *APEX2* (Bruker, 2008 ▶); cell refinement: *SAINT* (Bruker, 2008 ▶); data reduction: *SAINT*; program(s) used to solve structure: *SHELXS97* (Sheldrick, 2008 ▶); program(s) used to refine structure: *SHELXL97* (Sheldrick, 2008 ▶); molecular graphics: *ORTEP-3* (Farrugia, 1997 ▶); software used to prepare material for publication: *SHELXL97* and *PLATON* (Spek, 2003 ▶).

## Supplementary Material

Crystal structure: contains datablocks I, global. DOI: 10.1107/S1600536809003432/wn2308sup1.cif

Structure factors: contains datablocks I. DOI: 10.1107/S1600536809003432/wn2308Isup2.hkl

Additional supplementary materials:  crystallographic information; 3D view; checkCIF report

## Figures and Tables

**Table 1:** Hydrogen-bond geometry (Å, °)

*D*—H⋯*A*	*D*—H	H⋯*A*	*D*⋯*A*	*D*—H⋯*A*
C7—H7⋯O1^i^	0.93	2.63	3.272 (2)	127
C7—H7⋯O2^i^	0.93	2.63	3.553 (3)	172

Symmetry code: (i) 
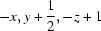
.

**Fig. 1.**
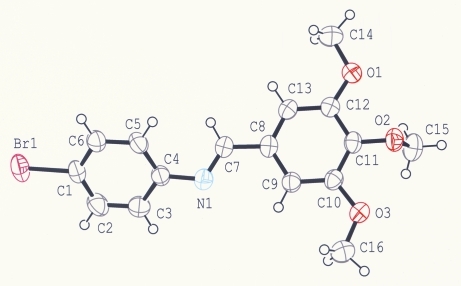
The molecular structure of the title compound, with the atom-labelling. Displacement ellipsoids of non-H atoms are drawn at the 50% probability level. Hydrogen atoms are denoted by spheres of arbitrary radius.

**Fig. 2.**
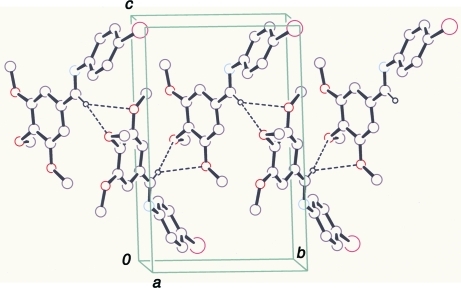
A partial packing diagram, viewed along the a axis. H atoms not involved in the C—H···O hydrogen bonds have been omitted.

## supplementary crystallographic information

### Comment

Recently, we reported two Schiff-base compounds with 4-bromoaniline units that have been structurally characterized (Khalaji *et al.*, 2007; Khalaji & Harrison, 2008). In continuation of these studies, the title compound was prepared and its structure has been determined.

An *ORTEP* plot, with the atomic numbering scheme is depicted in Fig. 1. The two benzene rings are twisted with respect to each other at an angle of 38.3 (1)°. In the crystal structure, the molecules are connected by weak bifurcated C—H···(O, O) hydrogen bonds, forming a helical chain along the *b* axis.

The C7═N1 bond length of 1.268 (3) Å conforms to the value for a double bond, and is slightly shorter than the corresponding bond length in *N*-(2-benzylidenepropylidene)-4-bromoaniline [C23═N23 1.288 (6) Å; Khalaji *et al.*, 2007] and *β*-phenylcinnamaldehyde-4-bromoaniline [C7═N1 1.277 (4) Å; Khalaji & Harrison, 2008]. The C4—N1 bond length of 1.421 (2) Å conforms to the value for a single bond, and, in turn, is slightly longer than the corresponding bond length in *N*-(2-benzylidenepropylidene)-4-bromoaniline [C24—N23 1.411 (7) Å] and *β*-phenylcinnamaldehyde-4-bromoaniline [C6—N1 1.407 (4) Å]. All other bond lengths in the three related Schiff-base compounds are quite similar. For the title compound, the torsion angle, C8—C7—N1—C4, is 179.20 (18)°, indicating a virtually planar *E*-configuration with respect to the imine C═N bond (Khalaji *et al.*, 2007; Khalaji & Harrison, 2008).

In comparison with the isotypic structure of C_16_H_16_ClNO_3_ (Dehno Khalaji *et al.*, 2009), all interatomic distances and angles (except those involving the halogen atom) are very similar.

### Experimental

The title compound was prepared by the reaction of 3,4,5-trimethoxybenzaldehyde (1 mmol, 0.196 g) and 4-bromoaniline (1 mmol, 0.172 g), which were dissolved in methanol (10 ml). The mixture was stirred at room temperature for 30 min. Colourless single crystals suitable for X-ray structure analysis were obtained by recrystallization from a methanol/chloroform (1:1 *v*/*v*) solution.

### Refinement

H atoms were positioned geometrically (C—H = 0.93 or 0.96 Å) and refined as riding, with *U*_iso_(H) = 1.2*U*_eq_(C) or 1.5*U*_eq_(methyl C), allowing for free rotation of the methyl groups.

### Crystal data

**Table:**

C_16_H_16_BrNO_3_	*F*(000) = 356
*M**_r_* = 350.21	*D*_x_ = 1.500 Mg m^−^^3^
Monoclinic, *P*2_1_	Mo *K*α radiation, λ = 0.71073 Å
Hall symbol: P 2yb	Cell parameters from 9957 reflections
*a* = 7.1951 (4) Å	θ = 2.9–29.0°
*b* = 8.3722 (5) Å	µ = 2.66 mm^−^^1^
*c* = 13.2882 (8) Å	*T* = 296 K
β = 104.413 (3)°	Block, colourless
*V* = 775.27 (8) Å^3^	0.40 × 0.30 × 0.15 mm
*Z* = 2	

### Data collection

**Table:**

Bruker APEXII CCD diffractometer	3497 independent reflections
Radiation source: fine-focus sealed tube	3064 reflections with *I* > 2σ(*I*)
graphite	*R*_int_ = 0.023
ω scans	θ_max_ = 28.0°, θ_min_ = 2.9°
Absorption correction: multi-scan (*SADABS*; Bruker, 2006)	*h* = −9→9
*T*_min_ = 0.403, *T*_max_ = 0.671	*k* = −10→10
18229 measured reflections	*l* = −17→17

### Refinement

**Table:**

Refinement on *F*^2^	Secondary atom site location: difference Fourier map
Least-squares matrix: full	Hydrogen site location: inferred from neighbouring sites
*R*[*F*^2^ > 2σ(*F*^2^)] = 0.025	H-atom parameters constrained
*wR*(*F*^2^) = 0.065	*w* = 1/[σ^2^(*F*_o_^2^) + (0.0339*P*)^2^ + 0.0173*P*] where *P* = (*F*_o_^2^ + 2*F*_c_^2^)/3
*S* = 1.09	(Δ/σ)_max_ = 0.002
3497 reflections	Δρ_max_ = 0.30 e Å^−^^3^
193 parameters	Δρ_min_ = −0.53 e Å^−^^3^
1 restraint	Absolute structure: Flack (1983), 1511 Friedel pairs
Primary atom site location: structure-invariant direct methods	Flack parameter: 0.012 (6)

### Special details

**Table:**

Geometry. All e.s.d.'s (except the e.s.d. in the dihedral angle between two l.s. planes) are estimated using the full covariance matrix. The cell e.s.d.'s are taken into account individually in the estimation of e.s.d.'s in distances, angles and torsion angles; correlations between e.s.d.'s in cell parameters are only used when they are defined by crystal symmetry. An approximate (isotropic) treatment of cell e.s.d.'s is used for estimating e.s.d.'s involving l.s. planes.
Refinement. Refinement of *F*^2^ against ALL reflections. The weighted *R*-factor *wR* and goodness of fit *S* are based on *F*^2^, conventional *R*-factors *R* are based on *F*, with *F* set to zero for negative *F*^2^. The threshold expression of *F*^2^ > σ(*F*^2^) is used only for calculating *R*-factors(gt) *etc*. and is not relevant to the choice of reflections for refinement. *R*-factors based on *F*^2^ are statistically about twice as large as those based on *F*, and *R*- factors based on ALL data will be even larger.

### Fractional atomic coordinates and isotropic or equivalent isotropic displacement parameters (Å^2^)

**Table:**

	*x*	*y*	*z*	*U*_iso_*/*U*_eq_	
Br1	0.99402 (3)	0.96811 (5)	0.988948 (16)	0.06694 (9)	
O1	−0.26888 (18)	0.4772 (2)	0.36904 (9)	0.0522 (3)	
O2	−0.46171 (19)	0.29368 (19)	0.46988 (11)	0.0498 (3)	
O3	−0.3668 (2)	0.2687 (2)	0.67538 (12)	0.0600 (4)	
N1	0.2656 (2)	0.6014 (2)	0.79047 (14)	0.0502 (4)	
C1	0.7715 (3)	0.8470 (2)	0.92769 (15)	0.0450 (4)	
C2	0.6245 (3)	0.8386 (3)	0.97682 (18)	0.0595 (6)	
H2	0.6355	0.8891	1.0404	0.071*	
C3	0.4614 (4)	0.7546 (3)	0.9308 (2)	0.0604 (6)	
H3	0.3634	0.7458	0.9648	0.072*	
C4	0.4402 (3)	0.6827 (2)	0.83465 (15)	0.0436 (4)	
C5	0.5939 (3)	0.6886 (3)	0.78856 (16)	0.0478 (5)	
H5	0.5850	0.6368	0.7256	0.057*	
C6	0.7594 (3)	0.7705 (3)	0.83532 (17)	0.0498 (5)	
H6	0.8618	0.7736	0.8043	0.060*	
C7	0.2013 (3)	0.6071 (2)	0.69260 (16)	0.0427 (4)	
H7	0.2700	0.6651	0.6542	0.051*	
C8	0.0246 (3)	0.5272 (2)	0.63686 (16)	0.0411 (4)	
C9	−0.0833 (3)	0.4364 (2)	0.69019 (15)	0.0432 (5)	
H9	−0.0454	0.4266	0.7621	0.052*	
C10	−0.2484 (3)	0.3611 (2)	0.63335 (16)	0.0430 (4)	
C11	−0.3059 (3)	0.3758 (2)	0.52599 (16)	0.0411 (4)	
C12	−0.1991 (2)	0.4695 (3)	0.47377 (12)	0.0403 (3)	
C13	−0.0336 (3)	0.5450 (2)	0.53043 (16)	0.0417 (4)	
H13	0.0381	0.6078	0.4963	0.050*	
C14	−0.1666 (4)	0.5679 (4)	0.3108 (2)	0.0785 (8)	
H14A	−0.1641	0.6779	0.3316	0.118*	
H14B	−0.2287	0.5591	0.2382	0.118*	
H14C	−0.0377	0.5284	0.3232	0.118*	
C15	−0.6378 (3)	0.3769 (4)	0.4588 (2)	0.0673 (7)	
H15A	−0.6588	0.3972	0.5262	0.101*	
H15B	−0.7412	0.3133	0.4189	0.101*	
H15C	−0.6324	0.4765	0.4238	0.101*	
C16	−0.3111 (5)	0.2366 (4)	0.7828 (2)	0.0761 (8)	
H16A	−0.1813	0.1983	0.8010	0.114*	
H16B	−0.3944	0.1569	0.7997	0.114*	
H16C	−0.3198	0.3327	0.8208	0.114*	

### Atomic displacement parameters (Å^2^)

**Table:**

	*U*^11^	*U*^22^	*U*^33^	*U*^12^	*U*^13^	*U*^23^
Br1	0.05738 (13)	0.06441 (15)	0.06520 (14)	−0.01188 (12)	−0.01083 (9)	−0.00513 (15)
O1	0.0556 (7)	0.0567 (8)	0.0431 (6)	−0.0113 (9)	0.0100 (5)	−0.0013 (9)
O2	0.0463 (7)	0.0467 (8)	0.0547 (8)	−0.0096 (7)	0.0092 (6)	−0.0106 (7)
O3	0.0588 (9)	0.0704 (10)	0.0506 (9)	−0.0236 (7)	0.0135 (7)	0.0033 (7)
N1	0.0478 (9)	0.0533 (10)	0.0498 (11)	−0.0107 (8)	0.0126 (8)	−0.0056 (8)
C1	0.0423 (9)	0.0395 (11)	0.0456 (11)	−0.0019 (8)	−0.0036 (8)	−0.0007 (8)
C2	0.0630 (13)	0.0664 (15)	0.0468 (12)	−0.0018 (11)	0.0094 (10)	−0.0168 (10)
C3	0.0582 (13)	0.0769 (18)	0.0500 (14)	−0.0086 (11)	0.0208 (11)	−0.0114 (10)
C4	0.0425 (9)	0.0441 (12)	0.0418 (10)	−0.0030 (9)	0.0060 (8)	−0.0009 (8)
C5	0.0463 (10)	0.0538 (14)	0.0400 (11)	−0.0014 (10)	0.0044 (8)	−0.0099 (9)
C6	0.0408 (10)	0.0595 (14)	0.0466 (12)	−0.0002 (9)	0.0060 (9)	−0.0020 (10)
C7	0.0376 (9)	0.0393 (10)	0.0504 (12)	−0.0019 (8)	0.0095 (8)	−0.0013 (8)
C8	0.0355 (9)	0.0337 (9)	0.0524 (11)	0.0014 (7)	0.0076 (8)	−0.0044 (7)
C9	0.0418 (9)	0.0433 (13)	0.0442 (9)	−0.0024 (7)	0.0101 (7)	−0.0026 (7)
C10	0.0434 (10)	0.0377 (11)	0.0497 (11)	−0.0038 (8)	0.0148 (8)	−0.0041 (8)
C11	0.0378 (9)	0.0345 (10)	0.0503 (11)	−0.0006 (8)	0.0097 (8)	−0.0080 (8)
C12	0.0412 (8)	0.0356 (8)	0.0449 (8)	0.0025 (11)	0.0122 (6)	−0.0028 (11)
C13	0.0397 (9)	0.0371 (10)	0.0502 (11)	−0.0020 (7)	0.0148 (8)	−0.0011 (8)
C14	0.0817 (17)	0.102 (2)	0.0505 (14)	−0.0239 (16)	0.0142 (13)	0.0169 (14)
C15	0.0429 (11)	0.0708 (17)	0.0828 (18)	−0.0044 (12)	0.0056 (11)	−0.0027 (13)
C16	0.0790 (17)	0.086 (2)	0.0611 (18)	−0.0228 (16)	0.0134 (15)	0.0180 (13)

### Geometric parameters (Å, °)

**Table:**

Br1—C1	1.8992 (19)	C7—C8	1.464 (3)
O1—C12	1.358 (2)	C7—H7	0.9300
O1—C14	1.414 (3)	C8—C9	1.399 (3)
O2—C11	1.367 (2)	C8—C13	1.379 (3)
O2—C15	1.421 (3)	C9—H9	0.9300
O3—C16	1.409 (3)	C10—C9	1.390 (3)
O3—C10	1.368 (2)	C11—C10	1.388 (3)
C1—C2	1.376 (3)	C12—C13	1.392 (3)
C1—C6	1.368 (3)	C12—C11	1.397 (3)
C2—H2	0.9300	C13—H13	0.9300
C3—C2	1.375 (3)	C14—H14A	0.9600
C3—H3	0.9300	C14—H14B	0.9600
C4—N1	1.421 (2)	C14—H14C	0.9600
C4—C3	1.386 (3)	C15—H15A	0.9600
C4—C5	1.392 (3)	C15—H15B	0.9600
C5—C6	1.380 (3)	C15—H15C	0.9600
C5—H5	0.9300	C16—H16A	0.9600
C6—H6	0.9300	C16—H16B	0.9600
C7—N1	1.268 (3)	C16—H16C	0.9600
			
O1—C12—C11	115.25 (16)	C6—C1—Br1	119.56 (16)
O1—C12—C13	125.52 (17)	C6—C1—C2	121.14 (18)
O1—C14—H14A	109.5	C6—C5—C4	120.69 (19)
O1—C14—H14B	109.5	C6—C5—H5	119.7
O1—C14—H14C	109.5	C7—N1—C4	117.70 (17)
O2—C11—C10	120.65 (17)	C8—C7—H7	118.3
O2—C11—C12	119.28 (17)	C8—C9—H9	120.7
O2—C15—H15A	109.5	C8—C13—C12	120.43 (18)
O2—C15—H15B	109.5	C8—C13—H13	119.8
O2—C15—H15C	109.5	C9—C8—C7	120.90 (18)
O3—C10—C9	124.70 (18)	C10—O3—C16	118.18 (19)
O3—C10—C11	114.39 (17)	C10—C9—C8	118.58 (18)
O3—C16—H16A	109.5	C10—C9—H9	120.7
O3—C16—H16B	109.5	C10—C11—C12	120.00 (17)
O3—C16—H16C	109.5	C11—O2—C15	113.49 (18)
N1—C7—C8	123.41 (18)	C11—C10—C9	120.91 (17)
N1—C7—H7	118.3	C12—O1—C14	118.41 (17)
C1—C2—H2	120.4	C13—C8—C9	120.83 (17)
C1—C6—C5	119.45 (19)	C13—C8—C7	118.27 (17)
C1—C6—H6	120.3	C13—C12—C11	119.23 (16)
C2—C1—Br1	119.30 (15)	C12—C13—H13	119.8
C2—C3—C4	121.2 (2)	H14A—C14—H14B	109.5
C2—C3—H3	119.4	H14A—C14—H14C	109.5
C3—C2—C1	119.1 (2)	H14B—C14—H14C	109.5
C3—C2—H2	120.4	H15A—C15—H15B	109.5
C3—C4—N1	118.17 (18)	H15A—C15—H15C	109.5
C3—C4—C5	118.22 (19)	H15B—C15—H15C	109.5
C4—C3—H3	119.4	H16A—C16—H16B	109.5
C4—C5—H5	119.7	H16A—C16—H16C	109.5
C5—C4—N1	123.56 (17)	H16B—C16—H16C	109.5
C5—C6—H6	120.3		
			
Br1—C1—C2—C3	178.2 (2)	C6—C1—C2—C3	−1.4 (4)
Br1—C1—C6—C5	−177.04 (17)	C7—C8—C9—C10	−179.05 (17)
O1—C12—C11—O2	3.6 (3)	C7—C8—C13—C12	179.05 (18)
O1—C12—C11—C10	−179.35 (19)	C8—C7—N1—C4	179.19 (18)
O1—C12—C13—C8	−179.36 (19)	C9—C10—O3—C16	−5.2 (3)
O2—C11—C10—O3	−4.2 (3)	C9—C8—C13—C12	−1.5 (3)
O2—C11—C10—C9	175.96 (17)	C11—C10—O3—C16	174.9 (2)
O3—C10—C9—C8	179.93 (18)	C11—C12—C13—C8	0.2 (3)
N1—C4—C3—C2	−178.3 (2)	C11—C10—C9—C8	−0.2 (3)
N1—C4—C5—C6	179.6 (2)	C12—C11—C10—O3	178.83 (19)
N1—C7—C8—C13	178.51 (19)	C12—C11—C10—C9	−1.0 (3)
N1—C7—C8—C9	−0.9 (3)	C13—C8—C9—C10	1.5 (3)
C2—C1—C6—C5	2.6 (3)	C13—C12—C11—O2	−175.99 (19)
C3—C4—N1—C7	144.8 (2)	C13—C12—C11—C10	1.0 (3)
C3—C4—C5—C6	−3.0 (3)	C14—O1—C12—C11	−179.1 (2)
C4—C3—C2—C1	−2.0 (4)	C14—O1—C12—C13	0.5 (3)
C4—C5—C6—C1	−0.3 (4)	C15—O2—C11—C10	88.3 (2)
C5—C4—C3—C2	4.2 (4)	C15—O2—C11—C12	−94.7 (2)
C5—C4—N1—C7	−37.8 (3)		

### Hydrogen-bond geometry (Å, °)

**Table:**

*D*—H···*A*	*D*—H	H···*A*	*D*···*A*	*D*—H···*A*
C7—H7···O1^i^	0.93	2.63	3.272 (2)	127
C7—H7···O2^i^	0.93	2.63	3.553 (3)	172

Symmetry codes: (i) −*x*, *y*+1/2, −*z*+1.
